# Classical Model of Quons

**DOI:** 10.3390/e21090841

**Published:** 2019-08-27

**Authors:** Giorgio Kaniadakis, Antonio M. Scarfone

**Affiliations:** 1 Dipartimento di Scienza Applicata e Tecnologia, Politecnico di Torino, Corso Duca degli Abruzzi 24, 10129 Torino, Italy; 2 Istituto dei Sistemi Complessi, Consiglio Nazionale delle Ricerche (ISC-CNR), c/o Politecnico di Torino, Corso Duca degli Abruzzi 24, 10129 Torino, Italy

**Keywords:** classical model of boson and fermion statistics, inclusion/exclusion principle, nonlinear Fokker-Planck equation, type I quon statistics, type II quon statistics

## Abstract

By using the kinetic interaction principle, the quons statistics in the framework of kinetic theory is introduced. This is done by properly generalizing the inclusion/exclusion principle of standard boson and fermion statistics within a nonlinear classical model. The related nonlinear Fokker-Planck equation is introduced and the corresponding steady distribution describing quons statistics of type I and type II is derived.

## 1. Introduction

As is well known, one of the most fundamental theorems in quantum field theory and in quantum statistical mechanics, at the basis of many physical and chemical phenomena, is the spin-statistics theorem stated by Pauli [1,2].

This theorem fixes the statistical behavior of a many-body quantum system according to its spin. Bosons are integer spin particles whose creation and annihilation operators obey bilinear commutation relations. Their many-body wave function is symmetrical and the occupation number of particle in a given state is unlimited. On the opposite side, fermions are half-integer spin particles whose creation and annihilation operators obey bilinear anti-commutation relations. Their many-body wave function is anti-symmetric and the occupation number of the particle in a given state can never exceed the unity.

Experimental tests [3,4,5] have placed a straight limit to the possible violations of this theorem so that, today, it is widely accepted that elementary particles can be only bosons or fermions.

However, statistical deviations from bosons or fermions can be observed in quasi-particle excitations that occur in various condensed matter systems. Therefore, the study of physical systems that obey non-conventional statistics is one of the pillars of contemporary statistical physics [6,7,8,9,10,11,12]. Their interest spans from the theoretical foundation of generalized statistical mechanics [13,14], Fermi gas superfluid [15], high-temperature gas [16] and high-$T_{c}$ superconductivity [17], Laughlin particle with fractional charge related to fractional quantum Hall effect [18,19], Josephson junctions [20] and applications to quantum computation [21,22].

Basically, there are two different approaches to introduce non-conventional statistics. The first one is by modifying the bilinear algebraic relation between creation and annihilation operators and therefore the exchange factor between permuted particles. The second is by modifying the number of possible ways to put particles in a collection of single-particles state.

Within the first method we find: parastatistics [23], obtained by generalizing, respectively, the bilinear commutative and anti-commutative relation of creation annihilation operators in trilinear relation for para-Boson and para-Fermion; quons statistics of type I [24,25], with asymmetric *q*-numbers and type II [26,27], with symmetric *q*-numbers, obtained in the framework of *q*-deformed harmonic oscillator based on *q*-calculus [28]; fractional statistics of anyons [29,30,31] that are topological bi-dimensional quasi-particles derived in the framework of quantum groups arising from the study of quantum integrable systems and Yang-Baxter equation; Majorana fermions quasiparticles [32]; and Gentilionic statistics [33], obtained by using the permutation group theory with the indistinguishability principle of identical particles in the framework of non relativistic quantum mechanics.

Differently, within the second approaches we find: Haldane-Wu statistics [34,35], including semion [36], obtained by generalizing Pauli exclusion principle; intermediate statistics by Gentile [37], derived in a thermodynamical context by assuming that the maximum occupation particle number of an energy level is between one (standard fermions) and infinity (standard bosons); and more recently the interpolating boson-fermion statistics [38,39,40,41,42], used to study non relativistic quantum systems that obey to a generalized inclusion/exclusion principle, obtained by modifying the dependency of the transition probability from the occupation particle number of the starting and the arriving site [43,44].

In this paper, we propose an algebraic approach to introduce non-conventional statistics within a semiclassical kinetic framework. Following the kinetic interaction principle proposed in [43], which fixes the form of the transition probability $\pi (t,\;v_{i}\to v_{i+1})$ in such a way to take into account its dependence on the particle population of the starting site $a(f_{i})$ and of the arrive site $b(f_{i+1})$, we obtain a nonlinear Fokker-Planck equation describing the particle evolution.

In standard boson and fermion statistics, the inclusion/exclusion principle is taken into account by the relation

(1) $$\begin{array}{c} b(f)\mp a(f)=1\;. \end{array}$$

In fact, when, a(f)=f is fixed, it follows b(f)=1±f, and the Bose (+) and Fermi (-) factors are obtained.

The relation (1) can be generalized in

(2) $$\begin{array}{c} b(f)◯\;\mp \;a(f)=1\;, \end{array}$$

where x◯±y is a deformed composition law, depending on a deformation parameter ξ, and is reduced to the standard sum and subtraction in a suitable limit $\xi \to \xi_{0}$. Equation (2) defines the functional dependence between a(f) and b(f), and fixes the steady particle distribution. The relationship between the generalized composition law x◯±y and the induced statistics is the main goal of this work.

The plane of the paper is as follows. In the next Section 2, we briefly recall the kinetic interaction principle used to introduce the nonlinear Fokker-Planck equation governing the time evolution of the particles system toward the equilibrium. Section 3 contains our main result. Non-conventional statistics are introduced by means of a generalized composition law between the functions a(f) and b(f), which introduces nonlinear terms in the Fokker-Planck equation and then modifies substantially its steady state solution. Several well known statistics can be easily derived within this algebraic approach and new statistics can be obtained through the introduction of suitable composition laws. This is discussed in the subsequent Section 4 and Section 5, which introduce, within the present formalism, type II quons statistics and type I quons statistics, respectively. In the last Section 6 we report our conclusions.

## 2. The Exclusion-Inclusion Principle

Let us consider a classical stochastic Markovian process in the *n*-dimensional velocity space. It is described by the distribution function f≡f(t,v) which obeys the Pauli master equation

(3) $$\begin{array}{c} \frac{\partial \;f}{\partial \;t}=\int \left[\pi \left(t,\;v^{\prime }\to v\right)-\pi \left(t,\;v\to v^{\prime }\right)\right]\;d^{n}v^{\prime }\;. \end{array}$$

According to the kinetic interaction principle the transition probability $\pi (t,\;v\to v^{\prime })$, from the site *v* to the site $v^{\prime }$, can be written in

(4) $$\begin{array}{c} \pi \left(t,\;v\to v^{\prime }\right)=W\left(t;\;v,\;v^{\prime }\right)\;a\left(f\left(v\right)\right)\;b\left(f\left(v^{\prime }\right)\right)\;. \end{array}$$

This quantity defines a special interaction between the particles of the system that involve, separately and/or together, the two-particle bunches entertained at the start and arrival sites. It is factorized into the product of three terms. The first term $W(t;\;v,\;v^{\prime })$ is the transition rate that depends on the nature of the interaction between the particles and the bath, and is a velocity function of the starting *v* and arrival $v^{\prime }$ sites. The second factor a(f)≡a(f(v)) is an arbitrary function of the particle population of the starting site and satisfies the condition a(0)=0 because, if the initial site is empty, the transition probability is equal to zero. Without loss of generality, we can always impose on a(f) the further condition a(1)=1 by re-scaling the function a(f) and opportunely redefining the quantity $W(t;\;v,\;v^{\prime })$. The last factor b(f)≡b(f(v)) is an arbitrary function of the arrival site population and satisfies the condition b(0)=1 since the transition probability does not depend on the arrival site if, in it, particles are absent. The expression of the function b(f) plays a very important role as it allows us to introduce a sort of inclusion/exclusion effect in the system stimulating or inhibiting the transition to the arrival site, as a consequence of the interactions originated from collective effects.

Accounting for Equation (4), by using the Kramer-Moyal expansion, the Pauli master equation can be transformed in the following Fokker-Planck equation

(5) $$\begin{array}{c} \frac{\partial \;f}{\partial \;t}=\nabla \cdot \left[D\;a\left(f\right)\;b\left(f\right)\;\nabla \left(\beta \;U+\ln\frac{a(f)}{b(f)}\right)\right]\;, \end{array}$$

where $\nabla \equiv (\partial /\partial v_{1},\;\ldots ,\;\partial /\partial v_{n})$ is the gradient operator in the velocity space, *D* is the diffusion coefficient, $\beta =1/k_{B}\;T$ the inverse temperature and $U=\frac{1}{2}\;m\;v^{2}$ is the single particle kinetic energy.

Equation (5) can be rewritten in

(6) $$\begin{array}{c} \frac{\partial \;f}{\partial \;t}=\nabla \cdot \left(D\;m\;\beta \;\gamma (f)\;v+D\;\Omega (f)\;\nabla f\right)\;, \end{array}$$

where

(7) $$\begin{array}{c} \gamma (f)=a(f)\;b(f)\;, \end{array}$$

affects the drift current $j_{\mathrm{drift}}=D\;m\;\beta \;\gamma \left(f\right)\;v$, while

(8) $$\begin{array}{c} \Omega \left(f\right)=b\left(f\right)\;\frac{\partial \;a(f)}{\partial \;f}-a\left(f\right)\;\frac{\partial \;b(f)}{\partial \;f}\;, \end{array}$$

models the diffusion current $j_{\mathrm{diff}}=D\;\Omega \left(f\right)\;\nabla f$. The functions γ(f) and Ω(f) are scalar quantities depending only on f(t,v). In this way, both drift and diffusion current depend, in a nonlinear manner, on the distribution function through the population of the starting and arrival site.

The stationary distribution of the system described by Equation (5) follows from the condition

(9) $$\begin{array}{c} \frac{\partial \;f}{\partial \;t}=0\;, \end{array}$$

that, without loss of generality, implies the reletion

(10) $$\begin{array}{c} \beta \;U+\ln\frac{a(f)}{b(f)}=\beta \;\mu \;, \end{array}$$

where μ is a constant. This last, can be rewritten in

(11) $$\begin{array}{c} \frac{a(f)}{b(f)}=e^{-\epsilon }\;. \end{array}$$

which defines implicitly the statistical distribution of the steady state of the system, where ϵ=β(U−μ), with μ the chemical potential fixed by the normalization of the distribution function.

As well known, Fokker-Planck equation (5) is strictly related to a generalized entropic form, as discussed in [43], so that the steady state obtained from Equation (11) can also be obtained starting from an optimizing program performed to the corresponding entropic form.

## 3. Generalized Exclusion-Inclusion Principle

The kinetics of already known statistics can be derived from ansatz (4) by choosing opportunely the functions a(f) and b(f). For instance, the bosons and fermions statistics follow, in the quasi-classical picture, by posing

(12) $$\begin{array}{c} a(f)=f\;, \end{array}$$

(13) $$\begin{array}{c} b(f)=1\pm f\;. \end{array}$$

The corresponding Fokker-Planck equation for quasi-classical Bose and Fermi particles reads

(14) $$\begin{array}{c} \frac{\partial \;f}{\partial \;t}=\nabla \cdot \left(D\;m\;\beta \;f\;(1\pm f)\;v+D\;\nabla f\right)\;, \end{array}$$

that describes a drift-diffusion process with a constant diffusive current, being Ω(f)=1, and a nonlinear drift term. The corresponding steady state

(15) $$\begin{array}{c} f\left(\epsilon \right)=\frac{1}{e^{\epsilon }\mp 1}\;, \end{array}$$

follows by solving Equation (11) with the position (12) and (13).

To go one step further and introduce more general statistics, let us observe that Equation (13) actually may be rewritten in the two equivalent forms

(16) $$\begin{array}{c} b(f)=1\pm a(f)\;, \end{array}$$

or, alternatively

(17) $$\begin{array}{c} b(f)=a(1\pm f)\;. \end{array}$$

However, it is easy to see that, although both of these formulations are equivalent for a(f)=f, for another choice of a(f) different from the identity, Equations (16) and (17) in general do not coincide.

To overcome this problem and have a consistent relationship between the functions a(f) and b(f), we introduce a generalized operation x⊕y to denote a new composition law between real numbers, hereinafter named deformed sum. It depends on a deformation parameter ξ such that the generalized sum ⊕ reduces to the ordinary sum + in a suitable limit $\xi \to \xi_{0}$, that is x⊕y→x+y.

Reasonably, a deformed sum x⊕y should preserve the main proprieties of standard sum like commutativity, x⊕y=y⊕x; associativity x⊕(y⊕z)=(x⊕y)⊕z; the existence of neutral element $x⊕0^{*}=0^{*}⊕x=x$; the opposite $x⊕\bar{x}=\bar{x}⊕x=0^{*}$, where, in general, $0^{*}\neq 0$ and $\bar{x}\neq -x$. Equipped with these properties, the algebraic structure A≡(⊕:ℜ×ℜ→ℜ) forms an Abelian group.

In this way, a deformed subtraction can be introduced as the inverse operation of the deformed sum, that is $x⊖y\equiv x⊕\bar{y}$.

Within the algebra A, we can generalize Equations (16) and (17) in

(18) $$\begin{array}{c} b(f)=1◯\;\pm \;a(f)\;, \end{array}$$

(19) $$\begin{array}{c} b(f)=a(c\pm f)\;, \end{array}$$

where *c* is a constant depending on the deformation parameter that must reduce to the identity in the $\xi \to \xi_{0}$ limit.

Consistently, by requiring that both Equations (18) and (19) define the same function b(f) for any choice of a(f), we obtain the following functional equation

(20) $$\begin{array}{c} 1◯\;\pm \;a(f)=a(c\pm f)\;. \end{array}$$

Thus, the generalized composition ◯± fixes univocally the function a(f). In fact, by posing

(21) $$\begin{array}{c} x◯\;\pm \;y=a\left(a^{-1}\left(x\right)\pm a^{-1}\left(y\right)\right)\;, \end{array}$$

we easily realize that Equations (18) and (19) coincide each to the other whenever we choose $c=a^{-1}\left(1\right)$.

According to definition (21), the neutral element is given by $0^{*}\equiv a\left(0\right)$ while the opposite is $\bar{x}\equiv a\left(-a^{-1}\left(x\right)\right)$.

Clearly, Equation (20) imposes a(f), and then b(f), to depend on the deformation parameter too so that, as expected in the $\xi \to \xi_{0}$ limit, these functions behave like a(f)→f and b(f)→1±f, respectively.

Notice also that ansatz (21) requires that a(x) be a monotonic, and then invertible, function at least in the range [0,1] of a distribution function.

Based on the algebra A, we can introduce several relevant generalized functions. Among them, the generalized exponential $E\left(x\right)\in {ℜ}^{+}$, defined in

(22) $$\begin{array}{c} E\left(x\right)=\exp\left(a^{-1}\left(x\right)\right)\;, \end{array}$$

that satisfies the relation

(23) $$\begin{array}{c} E(x⊕y)=E(x)\;E(y)\;, \end{array}$$

as well as its inverse function, the generalized logarithm L(x) for $x\in {ℜ}^{+}$, with L(E(x))=E(L(x))=x, defined in

(24) $$\begin{array}{c} L\left(x\right)=a\left(\ln(x)\right)\;, \end{array}$$

that satisfies the dual relation

(25) $$\begin{array}{c} L(x\;y)=L(x)⊕L(y)\;. \end{array}$$

In the $\xi \to \xi_{0}$ limit, in which ⊕ reduces to the standard sum, both the functions E(x) and L(x) reduce to the standard exponential and logarithm, respectively and Equations (23) and (25) reproduce the well know algebraic relation of standard exponential and logarithm functions.

## 4. Type II Quons

Within the formalism introduced above, let us now derive some statistics starting from deformed algebras already proposed in literature.

To start with, let us consider the κ-sum [43] defined in

(26) $$\begin{array}{c} x⊕y=x\;\sqrt{1+\kappa^{2}\;y^{2}}+y\;\sqrt{1+\kappa^{2}\;x^{2}}\;, \end{array}$$

whose deformation parameter ξ≡κ is limited to |κ|≤1 and the κ-sum recovers the standard sum in the κ→0 limit.

The above κ-sum is the momenta relativistic additivity law of special relativity and plays a central role in the construction of κ-statistical mechanics [45].

According to Equation (21), the function a(x) should be determined from the relation

(27) $$\begin{array}{c} a^{-1}\left(x⊕y\right)=a^{-1}\left(x\right)+a^{-1}\left(y\right)\;. \end{array}$$

In order to solve this functional equation we use the following identity

(28) $$\begin{array}{c} x=\frac{1}{\kappa }\;\sinh\left(\mathrm{arcsinh}\;(\kappa \;x)\right)\;, \end{array}$$

in the r.h.s. of Equation (26) that becomes

(29) $$\begin{array}{ccc} x⊕y & = & \frac{1}{\kappa }\;\sinh\left(\mathrm{arcsinh}\;(\kappa \;x)\right)\;\sqrt{1+{\left(\sinh\left(\mathrm{arcsinh}\;(\kappa \;y)\right)\right)}^{2}}\\ & & +\frac{1}{\kappa }\;\sinh\left(\mathrm{arcsinh}\;(\kappa \;y)\right)\;\sqrt{1+{\left(\sinh\left(\mathrm{arcsinh}\;(\kappa \;x)\right)\right)}^{2}}\\ & = & \frac{1}{\kappa }\;\sinh\left(\mathrm{arcsinh}\;(\kappa \;x)\right)\;\cosh\left(\mathrm{arcsinh}\;(\kappa \;y)\right)+\frac{1}{\kappa }\;\sinh\left(\mathrm{arcsinh}\;(\kappa \;y)\right)\;\cosh\left(\mathrm{arcsinh}\;(\kappa \;x)\right)\\ & = & \frac{1}{\kappa }\;\sinh\left(\mathrm{arcsinh}\;(\kappa \;x)+\mathrm{arcsinh}\;(\kappa \;y)\right)\;. \end{array}$$

This means that

(30) $$\begin{array}{c} \mathrm{arcsinh}\;(\kappa (x⊕y))=\mathrm{arcsinh}\;(\kappa \;x)+\mathrm{arcsinh}\;(\kappa \;y)\;. \end{array}$$

which forces us to define

(31) $$\begin{array}{c} a^{-1}\left(x\right)=\frac{1}{\kappa }\;\mathrm{arcsinh}\;\left(\kappa \;x\right)\;\Rightarrow \;a\left(x\right)=\frac{\sinh(\kappa \;x)}{\kappa }\;. \end{array}$$

It is worth observing that function a(x), derived in our approach within the κ-algebra, has already been studied in literature starting from [26,27] where quon statistics of type II has been introduced from a Hermitian version of the *q*-oscillator algebra of creation and annihilation operators.

In fact, recalling that algebra of type II quons is based on the symmetric *q*-numbers

(32) $$\begin{array}{c} \left[x\right]=\frac{q^{x}-q^{-x}}{q-q^{-1}}\;, \end{array}$$

which are invariant under the exchange q→1/q; it is easy to verify as definition (32) is related to function (31) according to

(33) $$\begin{array}{c} \left[x\right]=\frac{a(x)}{a(1)}\;. \end{array}$$

with κ=lnq.

Within the κ-algebra the generalized exponential reads $E\left(x\right)\equiv \exp_{\kappa }\left(x\right)$ and analogously the generalized logarithm reads $L\left(x\right)\equiv \ln_{\kappa }\left(x\right)$, where

(34) $$\begin{array}{c} \exp_{\kappa }\left(x\right)={\left(\sqrt{1+\kappa^{2}\;x^{2}}+\kappa \;x\right)}^{1/\kappa }\;, \end{array}$$

(35) $$\begin{array}{c} \ln_{\kappa }\left(x\right)=\frac{x^{\kappa }-x^{-\kappa }}{2\;\kappa }\;, \end{array}$$

and fulfill relations (23) and (25), respectively, with the κ-sum given in (26).

The deformed-subtraction is given in

(36) $$\begin{array}{c} x⊖y\equiv x\;\sqrt{1+\kappa^{2}\;y^{2}}-y\;\sqrt{1+\kappa^{2}\;x^{2}}\;, \end{array}$$

being, in this case, $0^{*}\equiv 0$ and $\bar{x}\equiv -x$.

The function a(f) given in Equation (31) defines univocally the function b(f) throughout Equations (18) or (19) with c=arcsinh(κ)/κ.

Therefore, the nonlinear kinetic underling type II quons statistics is depicted by a linear Fick diffusive current

(37) $$\begin{array}{c} j_{\mathrm{diff}}=D\;\nabla \;f\;, \end{array}$$

with a constant diffusive coefficient. In fact, it is straightforward to verify from Equation (8) that in this case Ω=1 [46]. Thus, like standard bosons and fermions, type II quons also undergo classical diffusive process governed by a linear diffusion current.

The corresponding nonlinear Fokker-Planck equation becomes

(38) $$\begin{array}{c} \frac{\partial \;f}{\partial \;t}=\nabla \left(D\;m\;\beta \;\gamma (f)\;v+D\;\nabla \;f\right)\;, \end{array}$$

where

(39) $$\begin{array}{c} \gamma \left(f\right)=\gamma_{{}_{+}}\;e^{2\;\kappa \;f}+\gamma_{{}_{-}}\;e^{-2\;\kappa \;f}+\gamma_{{}_{0}}\;, \end{array}$$

with

(40) $$\begin{array}{c} \gamma_{{}_{+}}=\frac{\pm \sqrt{1+\kappa^{2}}+\kappa }{4\kappa^{2}}\;,\;\gamma_{{}_{-}}=\frac{\pm \sqrt{1+\kappa^{2}}-\kappa }{4\kappa^{2}}\;,\;\gamma_{{}_{0}}=-\gamma_{{}_{+}}-\gamma_{{}_{-}}\;. \end{array}$$

The steady state follows from Equation (11), that in this case reads

(41) $$\begin{array}{c} \frac{\sinh(\kappa \;f)}{\sinh(\mathrm{arcsinh}(\kappa )\pm \kappa \;f)}=e^{-\epsilon }\;, \end{array}$$

that solved for f(ϵ) gives

(42) $$\begin{array}{c} f\left(\epsilon \right)=\frac{1}{\kappa }\;\mathrm{arctanh}\left(\frac{\kappa }{e^{\epsilon }\mp \sqrt{1+\kappa^{2}}}\right)\;. \end{array}$$

As easy check, in the κ→0 limit, functions a(f) and b(f) reduce to the one of standard bosons and fermions (12) and (13), as well as the nonlinear Fokker-Planck equation (38) reduces to Equation (14) and, in the same limit, the steady state (42) reproduces distribution (15).

## 5. Type I Quons

As known, type I quons firstly studied in [23], are based on the asymmetric version of *q*-numbers

(43) $$\begin{array}{c} {\left[x\right]}_{q}=\frac{q^{x}-1}{q-1}\;, \end{array}$$

that is strictly related to the *q*-calculus introduced by Jackson [28]. In the present context, type I quons can be derived starting from the following deformed sum

(44) $$\begin{array}{c} x⊕y=x+y+(q-1)\;x\;y\;, \end{array}$$

emerging also in the context of generalized statistical mechanics [47]. The deformation parameter ξ≡q, is restricted to q≥0 and the *q*-sum recovers the standard sum in the q→1 limit.

Equation (44) can be rewritten in

(45) $$\begin{array}{c} x⊕y=\frac{[1+(q-1)\;x]\;[1+(q-1)\;y]-1}{q-1}\;, \end{array}$$

so that

(46) $$\begin{array}{c} 1+(q-1)\;(x⊕y)=[1+(q-1)\;x]\;[1+(q-1)\;y]\;, \end{array}$$

which forces us to define the quantity

(47) $$\begin{array}{c} a^{-1}\left(x\right)=\frac{1}{\ln q}\;\ln\left(1+\left(q-1\right)\;x\right)\;, \end{array}$$

and its inverse a(x) coincides with function defined in Equation (43), that is

(48) $$\begin{array}{c} a\left(x\right)\equiv {\left[x\right]}_{q}\;. \end{array}$$

Asymmetric *q*-numbers have been employed in [23,24,25] to introduce quon statistics of type I starting from the *q*-generalization of the quantum oscillator algebras of creation and annihilation operators, like for the type II.

However, the algebra of asymmetric *q* numbers differs from those of the symmetric one since

(49) $$\begin{array}{c} {\bar{\left[x\right]}}_{q}=-q^{-x}\;{\left[x\right]}_{q}\;, \end{array}$$

being, in general $0^{*}=0$ and

(50) $$\begin{array}{c} \bar{x}=-\frac{x}{1+(q-1)\;x}\;, \end{array}$$

that is, the opposite in $A\equiv ({⊕}_{q},\;ℜ)$ does not coincides with the opposite of the ordinary algebras in *ℜ* like in the symmetric case.

Within the *q*-algebra the generalized exponential reads $E\left(x\right)\equiv \exp_{q}\left(x\right)$ and analogously the generalized logarithm reads $L\left(x\right)\equiv \ln_{q}\left(x\right)$ where

(51) $$\begin{array}{c} \exp_{q}\left(x\right)={\left(1+(q-1)\;x\right)}^{1/\ln q}\;, \end{array}$$

(52) $$\begin{array}{c} \ln_{q}\left(x\right)=\frac{q^{\ln x}-1}{q-1}\;, \end{array}$$

that are strictly related to the *q*-exponential and *q*-logarithm introduced in the generalized statistical mechanics [47] and fulfill relations (23) and (25), respectively, with the *q*-sum given in (44).

The function a(f) given in Equation (48) defines univocally the function b(f) throughout Equations (18) or (19) with c=1. However, in this case due to relation (49), we must separate the case of boson-like quons, with

(53) $$\begin{array}{c} b\left(f\right)=1+q\;{\left[f\right]}_{q}\;, \end{array}$$

from the case of fermion-like quons, with

(54) $$\begin{array}{c} b\left(f\right)=1-q^{1-f}\;{\left[f\right]}_{q}\;. \end{array}$$

### 5.1. Bosons-Like Quons

The kinetic of boson-like quons is depicted by the nonlinear Fokker-Planck equation

(55) $$\begin{array}{c} \frac{\partial \;f}{\partial \;t}=\nabla \left(D\;m\;\beta \;\gamma_{{}_{\mathrm{Bose}}}\left(f\right)\;v+D\;\Omega_{{}_{\mathrm{Bose}}}\left(f\right)\;\nabla \;f\right)\;, \end{array}$$

with a nonlinear drift term

(56) $$\begin{array}{c} \gamma_{{}_{\mathrm{Bose}}}\left(f\right)=\gamma_{{}_{2}}\;q^{2\;f}+\gamma_{{}_{1}}\;q^{f}+\gamma_{{}_{0}}\;, \end{array}$$

where

(57) $$\begin{array}{c} \gamma_{{}_{2}}=\frac{q}{{\left(q-1\right)}^{2}}\;,\;\gamma_{{}_{1}}=-\frac{1+q}{{\left(q-1\right)}^{2}}\;,\;\gamma_{{}_{0}}=-\gamma_{{}_{2}}-\gamma_{{}_{1}}\;, \end{array}$$

and a nonlinear diffusive term

(58) $$\begin{array}{c} \Omega_{{}_{\mathrm{Bose}}}\left(f\right)=\frac{\ln q}{q-1}\;q^{f}\;, \end{array}$$

that reduces to a constant $\Omega_{{}_{\mathrm{Bose}}}\left(f\right)\to 1$ in the q→1 limit. Therefore, differently from the symmetric case, Boson-like type I quons undergo classical diffusive process governed by a nonlinear diffusion current.

The steady state classical Boson-like quons follows from Equation (11) that in this case reads

(59) $$\begin{array}{c} \frac{q^{f}-1}{q^{1+f}-1}=e^{-\epsilon }\;, \end{array}$$

that solved for f(ϵ) gives

(60) $$\begin{array}{c} f_{{}_{\mathrm{Bose}}}\left(\epsilon \right)=\frac{1}{\ln q}\;\ln\frac{e^{\epsilon }-1}{e^{\epsilon }-q}\;. \end{array}$$

As expected, in the q→1 limit, the steady state of the Boson-like quons (60) reduces to the stationary distribution of bosons.

### 5.2. Fermions-Like Quons

The kinetic of fermion-like quons is depicted by the nonlinear Fokker-Planck equation

(61) $$\begin{array}{c} \frac{\partial \;f}{\partial \;t}=\nabla \left(D\;m\;\beta \;\gamma_{{}_{\mathrm{Fermi}}}\left(f\right)\;v+D\;\Omega_{{}_{\mathrm{Fermi}}}\left(f\right)\;\nabla \;f\right)\;, \end{array}$$

with a nonlinear drift term

(62) $$\begin{array}{c} \gamma_{{}_{\mathrm{Fermi}}}\left(f\right)=\gamma_{{}_{+}}\;q^{f}+\gamma_{{}_{-}}\;q^{-f}+\gamma_{{}_{0}}\;, \end{array}$$

where

(63) $$\begin{array}{c} \gamma_{{}_{+}}=-\frac{1}{{\left(q-1\right)}^{2}}\;,\;\gamma_{{}_{-}}=-\frac{q}{{\left(q-1\right)}^{2}}\;,\;\gamma_{{}_{0}}=-\gamma_{{}_{+}}-\gamma_{{}_{-}}\;, \end{array}$$

and a nonlinear diffusive term

(64) $$\begin{array}{c} \Omega_{{}_{\mathrm{Fermi}}}\left(f\right)=\omega_{{}_{0}}+\omega_{{}_{+}}\;q^{f}+\omega_{{}_{-}}\;q^{-f}\;, \end{array}$$

where

(65) $$\begin{array}{c} \omega_{{}_{0}}=\frac{2\;q\;\ln q}{{\left(q-1\right)}^{2}}\;,\;\omega_{{}_{+}}=-\frac{\ln q}{{\left(q-1\right)}^{2}}\;,\;\omega_{{}_{-}}=-\frac{q\;\ln q}{{\left(q-1\right)}^{2}}\;. \end{array}$$

and reduces to a constant $\Omega_{{}_{\mathrm{Fermi}}}\left(f\right)\to 1$ in the q→1 limit. Again, type I quons undergo classical diffusive process governed by a nonlinear diffusion current.

The steady state now follows from relation

(66) $$\begin{array}{c} \frac{q^{f}-1}{q^{1-f}-1}=e^{-\epsilon }\;, \end{array}$$

that solved for f(ϵ) gives

(67) $$\begin{array}{c} f_{{}_{\mathrm{Fermi}}}\left(\epsilon \right)=\frac{1}{\ln q}\;\ln\left(\frac{1-e^{-\epsilon }}{2}+\sqrt{{\left(\frac{1-e^{-\epsilon }}{2}\right)}^{2}+q\;e^{-\epsilon }}\;\right)\;. \end{array}$$

and reduces to the stationary distribution of fermion particles in the q→1 limit.

## 6. Conclusions

In this paper we have proposed an algebraic approach to study many body particle systems obeying a non-conventional statistics, in the semiclassical picture. A nonlinear Fokker-Planck equation, describing the kinetic of collectively interacting particles, has been obtained according to a kinetic interaction principle. The particle current is fixed by the two functions a(f) and b(f) that regulate the transition probability from the departing site to the arrival site in a way that depends only on the population of the initial and final sites, respectively. In this formalism, bosons-like and fermions-like particles follow from a very easy assumption on the function a(f) and b(f) by means of a generalized version of the inclusion/exclusion principle given by b(f)◯∓a(f)=1, with b(f)=a(c±f) for a generalized composition law that fixes the form of the functions a(f), and then b(f), and consequently fixes the steady particle distribution.

Following this approach, we have studied boson-like and fermion like quons of type II [26], whose underling algebra is related with the generalized sum (26), as well as boson-like and fermion like quons of type I [13], whose underlying algebra is defined by the generalized sum (44). It has been shown that the kinetic of type II quons is described by a nonlinear Fokker-Planck equation with a nonlinear drift current and a linear diffusive current like in the case of standard Bose and Fermi particles; otherwise, the kinetic of type I quons is described by a nonlinear Fokker-Planck equation with a nonlinear drift current and a nonlinear diffusive current.

Finally, let us remark that, following the same approach described in this work, several non conventional statistics in the classical picture can be obtained employing different composition laws. For instance, in addition to the κ-sum and the *q*-sum studied in this paper, other examples can be found in the framework of the generalized statistical mechanics [48,49].
